# Comparative efficacy and safety of regimens including ritonavir-boosted lopinavir or nevirapine in antiretroviral-naïve HIV-1-infected individuals

**DOI:** 10.1186/1758-2652-13-S4-P11

**Published:** 2010-11-08

**Authors:** A Pereira, JP Lopes da Cruz, C Carvalho, C Gonçalves, F Antunes

**Affiliations:** 1 Hospital de Santa Maria, Infectious diseases, Lisbon, Portugal; 2 University of Lisbon Faculty of Medicine, Lisbon, Portugal

## Purpose of the study

Ritonavir-boosted lopinavir (LPV/r) or nevirapine (NVP) combined with two nucleosides are recommended for first-line regimens in antiretroviral-naïve HIV-1 patients. There are few comparative studies between these different class-based regimens. Efficacy and safety may vary from randomized studies to actual clinical practice.

## Methods

We analyzed retrospective data from 167 HIV-1 infected antiretroviral-naïve individuals initiating LPV/r or NVP plus two nucleoside transcriptase reverse inhibitors (NRTI) (between 1999 and 2006), according to current guidelines.

## Summary of results

LPV/r was given to 46.7%, whereas 53.3% received NVP. Average patient age was 42 years (range, 19-80), 23.4% were women and 72.5% Caucasians. Co-infection with hepatitis viruses was present in 34.1% of all patients. The first most frequently used NRTI backbone was zidovudine-lamivudine (84.4% all patients; 38.3% LPV/r; 46.1% NVP). An alteration on NRTI backbone without study-drug discontinuation was permitted. There were no statistically significant differences between groups in the former baseline variables. Patients receiving a LPV/r-based regimen had, in average, lower baseline T CD4 cell counts (P=0.004, Mann-Withney) and a higher viral load (P<0.0001, Mann-Withney) compared with those receiving NVP. Early response to treatment was evaluated by the number of patients with a viral load decline >1.0 log10 after one month of treatment: 91.7% for LPV/r (n=33/36) and 77.1% for NVP (n=27/35). Undetectable viral load after one year of treatment was 79.3% for LPV/r (n=46/58) and 82.8% for NVP (n=48/58); with an increase in T CD4 cell count by 8.9 and 1.9-fold for LPV/r (n=46) and NVP (n=54), respectively (P=0.003). The overall number of patients that discontinued therapy before completing one year of treatment were, respectively for LPV/r and NVP, 17.9% (n=14/78) and 23.6% (n=21/89). Toxicity was the most referred reason for study-drug discontinuation in both groups. After one year of treatment, toxicity grade III/IV blood biochemistry analyzed values (serum transaminases, total cholesterol, HDL, LDL and triglycerides) were 10.2% (n=27/264) for LPV/r and 8.33% (24/288) for NVP, compared to 6.44% (n=17/264) and 7.99 % (n=23/288) on baseline.

## Conclusions

LPV/r seems to have a better early response and immunological improvement. However, excluding the early discontinuation of therapy due to toxicity, NVP seems to have a lesser toxicity impact in the long-term.

